# Temporal and Geographic Patterns of Urologic Conferences in the United States: A Descriptive Analysis From 2022 to 2025

**DOI:** 10.7759/cureus.113319

**Published:** 2026-07-24

**Authors:** Austin Erickson, Jennifer Griffith, Trushar Patel

**Affiliations:** 1 Department of Urology, University of South Florida Morsani College of Medicine, Tampa, USA; 2 Department of Urology, University of South Florida, Tampa, USA

**Keywords:** conference scheduling, continuing medical education, geographic distribution, medical conference, urology

## Abstract

Introduction: Professional conferences are key platforms for education, networking, and the dissemination of new research. However, little is known about the timing, duration, and geographic distribution of these events across the United States. Understanding these patterns can guide strategies to improve accessibility, enhance participation, and reduce scheduling conflicts.

Methods: The study was conducted at the University of South Florida, Morsani College of Medicine, Tampa, Florida. We identified urology conferences held between 2022 and 2025 using publicly available sources, including conference websites, online calendars, and major urologic event organizers (WJ Weiser & Associates, UroToday, Grand Rounds in Urology). For each, we recorded the event dates, frequency, and geographic location.

Results: Across the study period, 85 organizations held 293 conference events. The most frequent host states were Texas (31), California (30), Arizona (21), Colorado (21), and Florida (20). By American Urological Association section, the Western Section hosted the most events (76), followed by the Southeast (70) and South Central (66). The average conference duration increased from 3.08 days in 2022 to 3.25 days in 2025. October (171 total conference days) and September (129) were the busiest months, while July was the least busy (15).

Conclusions: Urology conferences in the United States are geographically concentrated in the Western, Southeastern, and South Central AUA sections, and predominantly scheduled in the fall. These trends highlight opportunities to redistribute events more evenly across regions and months, potentially improving access and participation.

## Introduction

Professional conferences play a crucial role in the field of urology, offering essential opportunities for continuing medical education (CME) and professional development in the field. They provide a platform for the presentation of new research, including novel surgical techniques and advancements in patient care [1]. In addition to their educational value, conferences foster interprofessional relationships, mentorship, and collaboration among both trainees and practicing urologists [2-3].

The importance of conferences in maintaining clinical competency and improving patient outcomes has been increasingly recognized. Participation in CME activities, including national and regional meetings, has been associated with improved physician knowledge, adherence to evidence-based practices, and, in some cases, measurable improvements in patient care [4-5]. However, attendance at these conferences often requires substantial financial investment, time away from clinical responsibilities, and travel, which may limit accessibility for certain individuals [6].

Despite the recognized value of urologic conferences, little is known about their logistical aspects, such as location, timing, and duration. Understanding these patterns is essential to optimizing scheduling, improving accessibility, reducing travel burdens, and enhancing participation. While conferences occur year-round across the United States and encompass a wide range of subspecialties, their timing and geographic distribution vary considerably. For attendees with limited institutional support, tight schedules, or geographic isolation, these variations can pose significant barriers [7]. Furthermore, frequent long-distance travel can contribute to both financial strain and a significant carbon footprint [8].

As the specialty navigates resource limitations and shifting educational priorities, there is an increasing need to evaluate how conference logistics can be optimized for equity, efficiency, and sustainability. This study examines the timing, duration, and geographic distribution of in-person urology conferences in the United States between 2022 and 2025, with the goal of informing more inclusive and strategic conference planning.

## Materials and methods

The study was performed at the University of South Florida, Morsani College of Medicine, Tampa, Florida, USA, in 2025. We identified urologic organizations that held conferences in the United States between 2022 and 2025 using publicly available online sources, including search engines, conference websites, online calendars, electronic flyers, and event programs. Supplemental information was obtained from major urologic event organizers, including WJ Weiser & Associates, UroToday, and Grand Rounds in Urology. Given that all information was publicly available, this study was deemed exempt from IRB approval. For each conference, we recorded the event dates, frequency of occurrence during the study period, and geographic location. The data were analyzed to determine the average conference duration, the number of conferences per state, and the distribution across American Urological Association (AUA) sections. Temporal trends were assessed by calculating the total number of conference days per individual month, which were subsequently grouped per season.

Conferences were examined at both the organization level and individual event level, with most organizations repeating an event each year during the study period. Organizations and events are summarized using frequencies and proportions as well as mean and standard deviation for event duration. Event duration is compared across other characteristics using Kruskal-Wallis tests. A significance level of 0.05 is used for all tests. Analysis was completed using IBM SPSS Statistics, version 30.0 (released 2024, IBM Corp., Armonk, NY).

## Results

A total of 85 distinct conference organizations were identified. These organizations consisted of 33 (38.8%) sub-specialties, 22 (25.9%) states, 22 (25.9%) general urologies, and eight (9.4%) AUA section conference organizations. Of these organizations, 55 (64.7%) held an event annually during the study period, 19 (22.4%) held an event three out of the four years evaluated, and six (7.1%) held only one event during the study period. Of note, the six society meetings that were held only one year were mostly sub-specialty organizations (five, 83.3%) with one general urology organization (one, 16.7%) (Table 1). Each AUA section held a conference annually.

**Table 1:** Characteristics of organizations. AUA: American Urological Association

Characteristics	Organizations N (%) (N = 85)
		Organization type	
AUA section	8 (9.4)
General	22 (25.9)
Specialty	33 (38.8)
State society	22 (25.9)
Number of events held in 2022-2025	
4	55 (64.7)
3	19 (22.4)
2	5 (5.9)
1	6 (7.1)

**Figure 1:**
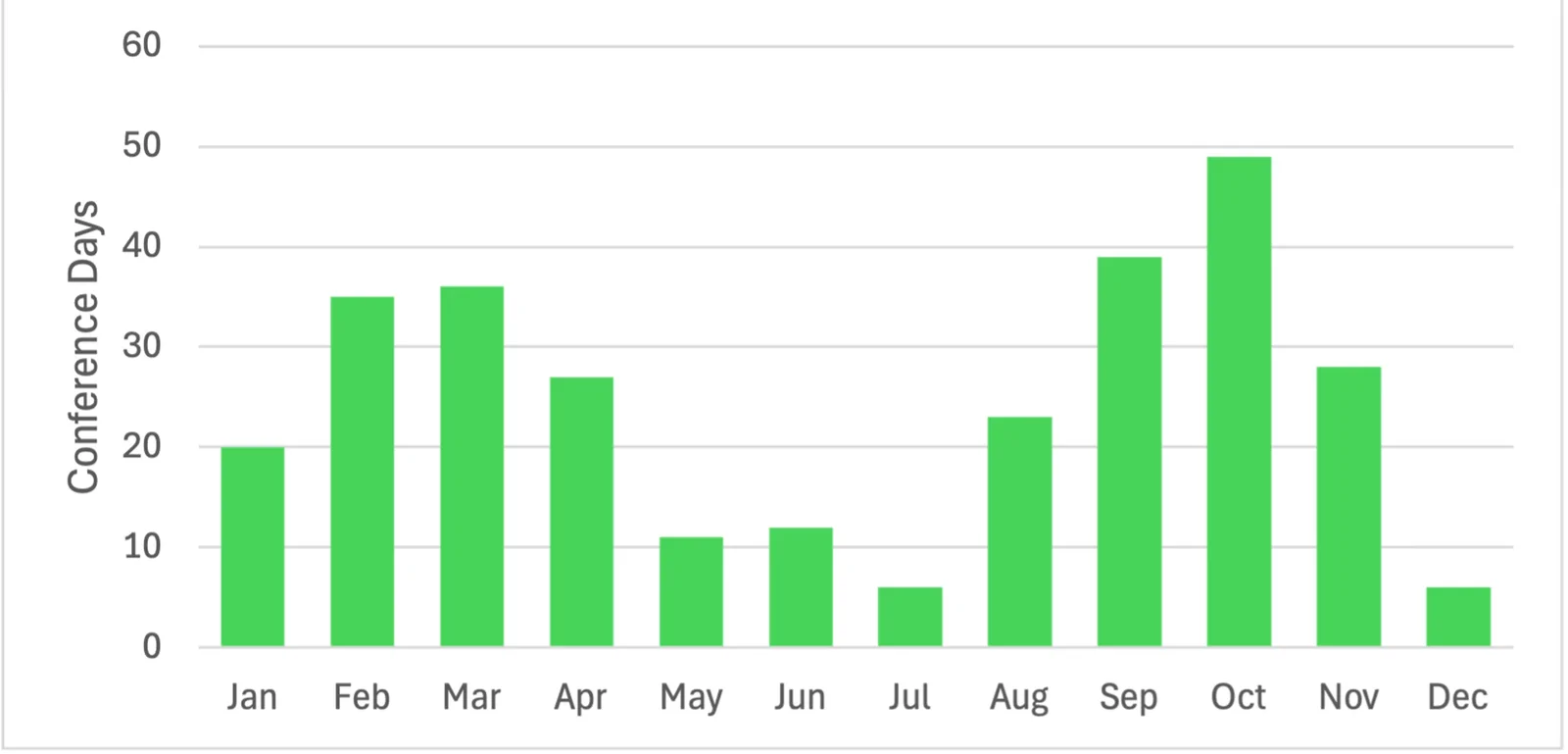
A column chart displaying the distribution of total conference days per month of urologic conferences from 2022 to 2025.

**Figure 2:**
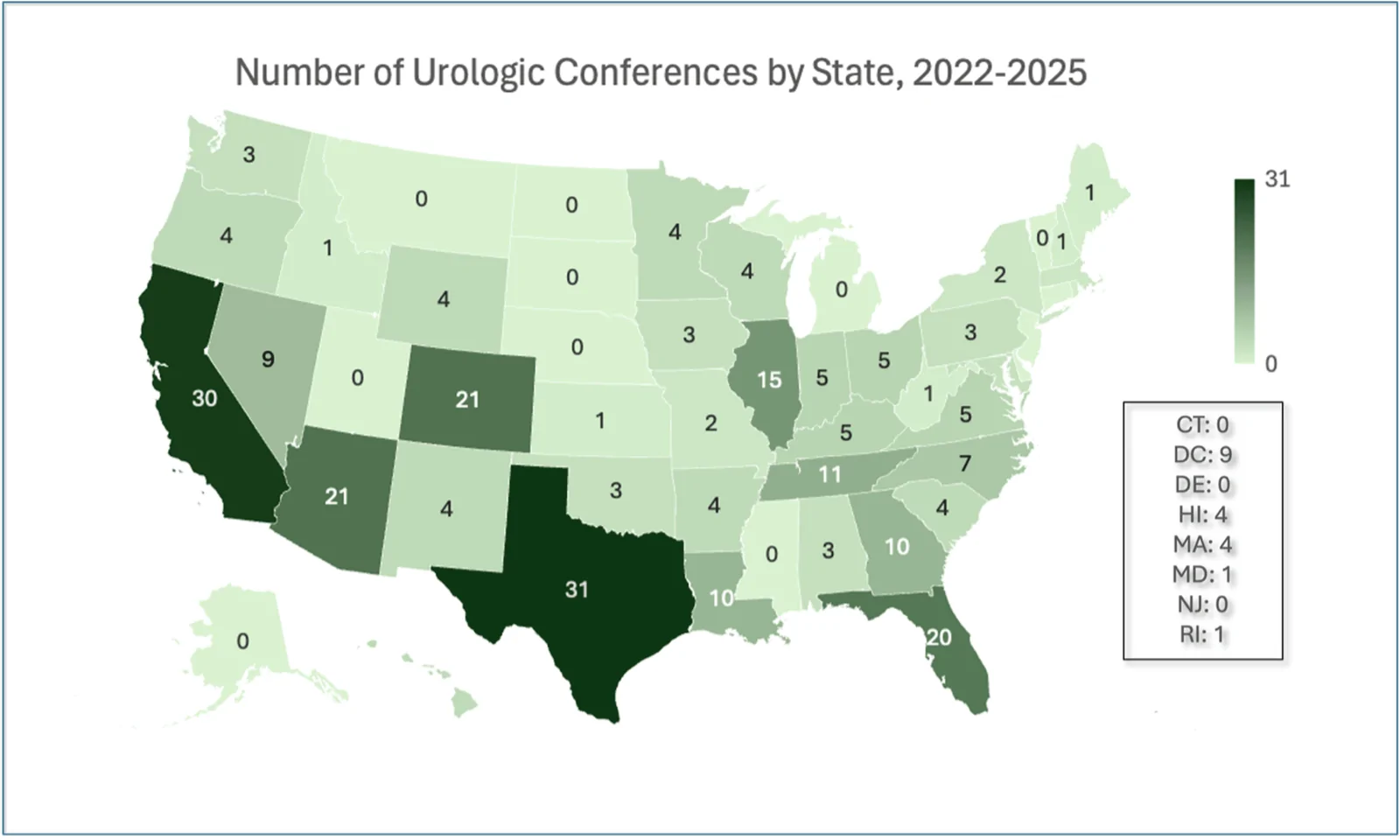
A heatmap of the United States displaying the number of urologic conferences by state from 2022 to 2025. Created using Microsoft Excel with Bing Maps add-in (version 2402, Microsoft Corporation, USA (2026)).

**Figure 3:**
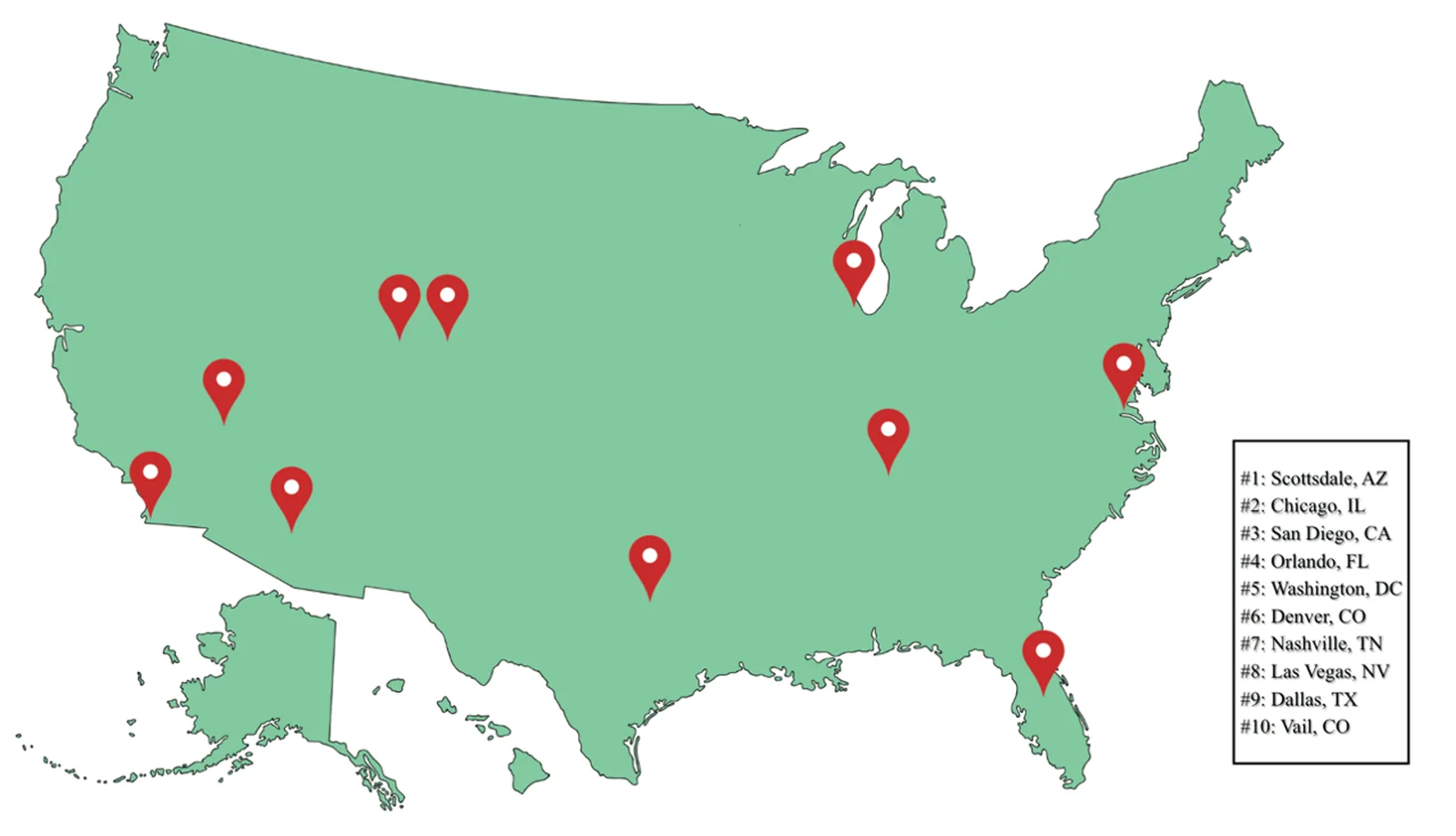
A map showing the geographic distribution of the top 10 cities in which conferences were held across the United States from 2022 to 2025 Created using Microsoft PowerPoint 2026 (Microsoft Corp., USA). The map was from the stock photos available on Microsoft PowerPoint.

Over the four years of interest, these 85 organizations held 293 events. The overall number of events is slowly increasing by year, with 64 in 2022, 71 in 2023, 79 in 2024, and 79 in 2025. Events were held more commonly in the fall with 116 (39.6%), followed by spring with 74 (25.3%), winter with 62 (21.2%), and, finally, summer with 41 (14.0%) (Table 2, Figure 1). The states with the highest numbers of events were Texas with 31 (10.6%), California with 30 (10.2%), Arizona with 21 (7.2%), Colorado with 21 (7.2%), and Florida with 20 (6.8%) (Figure 2). Twelve states did not host any urology conference events during the study period. When analyzed by AUA section, the Western Section held the most events (77), followed by the Southeast (73) and South Central (66) Sections. Conferences were often hosted in the same cities, most notably Scottsdale, Arizona (13), Chicago, Illinois (12), and San Diego, California (12) being among the top cities (Figure 3).

**Table 2:** Characteristics and duration of events. Kruskal-Wallis tests were used to determine the significance of the distribution of duration, as compared by various characteristics. A significance level of 0.05 was used for all tests.

Characteristics	Events N (%) (N = 293)	Duration (days)		
		Mean ± SD	Kruskal-Wallis χ² (df)	p-value
Organization type			79.26 (3)	<0.001
AUA section	32 (10.9)	4.22 ± 1.34		
General	77 (26.3)	3.01 ± 1.02		
Specialty	106 (36.2)	3.46 ± 1.02		
State society	78 (26.6)	2.29 ± 0.82		
Year			1.60 (3)	0.659
2022	64 (21.8)	3.08 ± 1.11		
2023	71 (24.2)	3.04 ± 1.22		
2024	79 (27.0)	3.13 ± 1.19		
2025	79 (27.0)	3.25 ± 1.17		
Season			16.92 (3)	0.001
Winter	62 (21.2)	3.08 ± 1.19		
Spring	74 (25.3)	2.72 ± 1.08		
Summer	41 (14.0)	3.17 ± 1.32		
Fall	116 (39.6)	3.37 ± 1.10		
Region			36.80 (5)	<0.001
Northeast US	28 (9.6)	3.07 ± 0.90		
Southeast US	73 (24.9)	2.89 ± 1.06		
South Central US	66 (22.5)	3.02 ± 0.94		
North Central US	36 (12.3)	2.53 ± 1.25		
Western US	77 (26.3)	3.37 ± 1.02		
International	13 (4.4)	5.15 ± 1.63		

The duration of the events ranged from one to seven days, with an average duration of 3.12 days (SD 1.17). The duration of events was significantly different by organization type (p < 0.001). Duration was the longest for AUA section meetings with an average (±SD) of 4.22 ± 1.34 days and lowest for state society meetings (2.29 ± 0.82 days). Event duration has not significantly changed over the four years (p = 0.659) but has shown a slight increase over time from 3.08 ± 1.11 days in 2022 to 3.25 ± 1.17 days in 2025. There was a significant difference in duration by season (p = 0.001), with fall events lasting the longest (3.37 ± 1.10 days) and spring events being the shortest (2.72 ± 1.08 days). There was a significant difference in event duration by region (p = 0.002 when excluding international events), with the north central region having significantly shorter events (2.53 ± 1.25 days) and the western region having the longest events (3.37 ± 1.02 days). When considering international events, they were significantly longer than all U.S. functions (5.15 ± 1.63 days; p < 0.001).

## Discussion

This study is the first to comprehensively identify and characterize trends in the geographic and temporal distribution of in-person urologic conferences within the United States during the post-COVID-19 period, from 2022 to 2025. The analysis reveals marked geographic concentration in a limited number of states, seasonal clustering in the fall, and a modest decline in conference duration. These patterns have important implications for accessibility, workforce development, and sustainability within the specialty. There was a significant concentration of conferences in a limited number of states, with Texas, California, Arizona, Colorado, and Florida hosting the highest amounts. The uneven geographic distribution, with 20 states hosting no events, may limit opportunities for trainees and practicing urologists in underserved regions, particularly medical students without a home urology program who rely on conferences for mentorship and career exploration. Given that early mentorship is a well-established factor in influencing students to pursue urology [9-11], expanding conference accessibility to underrepresented areas could be a valuable strategy for addressing the ongoing workforce shortage in the specialty [12,13]. This may also contribute to a widening disparity in the geographical distribution of urologists across the United States, as younger urologists tend to concentrate in urban metropolitan areas in a select few states [14]. While further research is required to determine the correlative relationship between the distribution of practicing urologists and the locations of urologic conferences, the inequity is notable.

Conference scheduling showed a clear preference for the fall, with October and September hosting most events, while summer (particularly July) was largely avoided. This clustering may align with academic calendars, vacation patterns, and seasonal weather, but it can also create overlap that limits attendance at multiple meetings. Strategies, such as consolidating similar events or distributing them more evenly throughout the year, could improve efficiency and accessibility, although each approach carries trade-offs in variety, flexibility, and logistics. The mean conference duration was approximately three days, showing a slight increase from 2022 to 2025. This trend may indicate a preference for longer conferences to increase opportunities for in-depth learning and networking. However, shorter conferences reduce cost and time away from clinical responsibilities, ultimately highlighting the importance of intentional planning to balance efficiency with the overall value of the conference experience.

Lastly, given the increasing awareness of environmental sustainability in healthcare, the geographic clustering of conferences raises concerns about the cumulative carbon footprint associated with participant travel. Conferences that require long-distance or air travel contribute more to greenhouse gas emissions as compared to shorter travel distances [8,15]. While specific data quantifying the environmental burden is currently lacking, the impact is likely substantial. Consolidating conferences to reduce overall travel demand and offering hybrid or virtual attendance options are potential strategies for minimizing this footprint and promoting more sustainable practices within the field. [16,17] However, consideration should also be made for how this impacts trainees, who use conferences as a means of networking and camaraderie. [2] Ultimately, there is viability to the use of virtual or hybrid conferences as an alternative method [18]; preferences of all attendees should be taken into account to ensure equity and accessibility to the largest number of participants.

The primary limitation of this study is its reliance on publicly available online data sources. As a result, conferences that were not well advertised or lacked an online presence may not have been captured. Additionally, the retrospective design limited data collection to conferences with accessible documentation, such as organizational webpages or recorded materials, potentially excluding less formally documented events. Although a comprehensive search strategy was employed, including the use of multiple sources to verify conference occurrences, these inherent limitations of the study design may have influenced the reported findings.

## Conclusions

This study provides a comprehensive analysis of the timing, location, and duration of urologic conferences held in the United States between 2022 and 2025. The results demonstrate clear patterns of geographic and seasonal clustering, reflecting scheduling preferences among organizing committees. These findings provide a foundation for future research aimed at exploring how these trends may impact conference attendance, accessibility, sustainability, educational value, and resource allocation within the field of urology.
